# Effect of Intranasal Ketamine on Pain Intensity after Cesarean Section: A Single-Center, Double Blind, Randomized Controlled Trial

**DOI:** 10.4314/ejhs.v33i1.8

**Published:** 2023-01

**Authors:** Abolfazl Firouzian, Nafiseh Faghani-Makrani, Zeinab Nazari, Mouna Faghani Ahangari

**Affiliations:** 1 Department of Anesthesiology, Faculty of Medicine, Mazandaran University of Medical Sciences, Sari, Iran; 2 Department of Obstetrics and Gynecology, Faculty of Medicine, Mazandaran University of Medical Sciences, Sari, Iran

**Keywords:** Pain, Postoperative, Analgesia, Administration, Intranasal, Ketamine, Cesarean Section

## Abstract

**Background:**

Although intravenous or intramuscular opioids are widely used for managing postoperative pain after cesarean section (CS), their side effects are bothering and limit their use. The aim of this study was to determine the effect of intranasal ketamine on pain intensity after CS.

**Methods:**

In a single-center, double-blind, parallel-group, randomized controlled trial, a total of 120 patients who were scheduled for elective CS were randomly assigned into two groups. After birth, 1 mg of midazolam was administered to all patients. In addition, 1 mg/kg intranasal ketamine was administered to patients in the intervention group. For patients in control group, normal saline was administered intranasally as a placebo. The severity of pain and nausea in the two groups was evaluated after 15, 30 and 60 minutes, as well as 2, 6 and 12 hours after the initial administration of the medications.

**Results:**

The trend of changes in pain intensity was decreasing and these changes were statistically significant (time effect; P<0.001). The pain intensity in the placebo group was higher than the intervention and the observed difference was statistically significant, regardless of the time studied (group effect; P<0.001). In addition, it was shown that regardless of the study group, the trend of changes in nausea severity was decreasing and these changes were statistically significant (time effect; P<0.001). Regardless of the time studied, the severity of nausea in the placebo group was higher than the intervention group (group effect; P<0.001).

**Conclusions:**

According to the results of this study, it seems that the using of intranasal ketamine (1 mg/kg), can be considered as an effective, well tolerated and safe method in reducing pain intensity as well as the need for postoperative opioid consumption after CS.

## Introduction

Cesarean section (CS) is one of the most common gynecological surgery, which due to various reasons such as increasing the age of marriage and socioeconomic status of society, its prevalence is increasing.It has been shown that the rates of CS have been increasing globally, as the sustained, unprecedented rise in CS rates is a major public health concern (1–2). Previously it has been stated that about 65% of Iranian pregnant women underwent cesarean section and about 22.5% of all deliveries in the world are performed by cesarean section (1–3).

After all surgeries, including CS, patients inevitably experience pain to varying degrees (3). The experience of pain in addition to causing discomfort for patients, can have adverse physiological effects such as non-clearance of respiratory secretions, ileus and reduce postoperative mobilization, which increases the risk of deep vein thrombosis and other complications and delays in postoperative recovery. In addition, acute postoperative pain after CS can be associated with longer mother-infant separation and interferes with successful breastfeeding immediately after birth, increased myocardial oxygen consumption and reactive hypoglycemia leading to delayed wound healing. Also, poor pain relief may even lead to chronic pain and increased postpartum depression (4–6). Therefore, finding a suitable way to provide a maximum pain relief and relaxation for patient with reduced complications is one of the highest priorities after CS and continues to be a relevant public health issue (7–9).

The search for the ideal method to manage postoperative pain is ongoing. So far, many studies has been performed for appropriate management of postoperative pain after CS. The multiplicity of studies and modalities indicates the lack of a clear and reliable method in reducing postoperative pain and shows that acute postoperative pain management after CS still remains a problem (10–11). At present, opioids, especially their injectable form, are widely used as the first line and gold standard modality in relieving acute postoperative pain after surgery, including CS (12, 13). On the other hand, pain is a multifactorial phenomenon that is not completely controlled by monotherapy with opioids (14–15). In addition, using opioids is usually associated with dose-dependent side effects such as respiratory depression, nausea, vomiting, urinary retention, pruritus, drowsiness, or postoperative ileus. Therefore, addition of adjuvant analgesics to opioids is a strategy that can lead to better pain control with fewer adverse effects (16–17).

Ketamine, an N-methyl-D-aspartate (NMDA) receptor antagonist, has been widely studied as an analgesic adjuvant for management of postoperative pain. Previous studies evaluate the efficacy of ketamine for managing postoperative pain and analgesic consumption after CS, with conflicting results (18–20). Ketamine has been previously used by various route of administration including intravenous, intramuscular, subcutaneous, sublingual and intranasal for pain management. However, administration of ketamine via the intra nasal route, especially in adults, has only recently been studied and requires further elucidation. Limited studies evaluated and confirmed the efficacy of intranasal ketamine for managing acute pain after tonsillectomy (21), spinal surgery (22), moderate or severe pain in emergency department (23), pre-hospital analgesia (24), and chronic pain (25).

Intranasal route of administration offers a variety of attractive options for local and systemic delivery of different medications. Intranasal administration is noninvasive, painless, easily administered, and relatively safe and is associated with a rapid onset of therapeutic effects and higher bioavailability due to avoid the first- pass effect (26–27). To the best of our knowledge there is no published study to evaluate the effect of intranasal ketamine for reducing postoperative pain after CS. Given the significant potential for a simple, safe and clinically and logistically convenient mode of analgesia for acute pain, defining intranasal ketamine's safety and efficacy seems especially important. Therefore, considering the abovementioned issues and the importance of relieving postoperative pain after CS and also limited available evidence, this study aimed to evaluate the effect of intranasal ketamine on pain intensity after CS. We hypothesized that using intranasal ketamine could provide additional pain relief on pain intensity after CS.

## Methods

In a prospective, single-center, double-blind, parallel-group, randomized controlled clinical trial a total of 120 women in Imam Khomeini hospital in Sari, Mazandaran province, north of Iran, were evaluated. The study was carried out between December 2021 and April 2022. Pregnant women with class I or II of American Society of Anesthesiologists, who scheduled for elective CS via Pfannenstiel incision under spinal anesthesia were included in this study. The study participants were included using a systematic random sampling method from consecutive patients.

Pre-anesthetic evaluation was done in the morning on the day of surgery, and eligibility criteria were checked before recruiting the study participants. Inclusion criteria were non-emergency CS, age between 20–35 years, to give a birth by CS between 37–40 weeks, no history of cardiovascular disease and hypertension, no history of mental disorders, no allergy to ketamine, and no history of opium addiction. Exclusion criteria were patients' unwillingness to participate or discontinue the study at any time during the study period, prolongation of CS duration (more than 1.5 hours), increased length of the incision for any reason, have a severe systemic diseases and the occurrence of any unusual complication during surgery or neonatal complications. Also, patients who had a history of alcohol abuse, were using opioids or psychotropic medication, who had a history of chronic pain disorder, with cognitive impairment, and with preeclampsia or were unable to understand the informed consent form were excluded.

Patients who fulfilled the inclusion criteria were randomly allocated into two equally sized groups of A and B (n=60) by a nurse anesthetist who was blind to the study groups, using sealed envelope technique and computer generated random numbers. Before surgery, all patients were instructed to use the patient-controlled analgesia (PCA) device, as well as rating the intensity of their pain, nausea, and post-dural puncture headache, using the visual analog scale (VAS; where 0 denotes to the least and 10 to the worst imaginable intensity), postoperatively. VAS is a self-reported tool and is not dependent on the researcher. In operating room, all patients were administered the same anesthetic protocol.

After birth and separation of umbilical cord and placenta, 1 mg of midazolam was administered to all patients. In addition, 1 mg/kg ketamine was administered intranasally to patients in the intervention group (group B). Normal saline was administered intranasally as a placebo for the control group (group A). To prepare the medications, the desired dose of the ketamine (1 mg/kg) added to the normal saline (total volume of 2 ml) and 1 ml of this solution was administered in each nostril. The control group was also given 2 ml of normal saline (1 ml per nostril). The syringes were numbered as 1 and 2 and were similar in terms of volume and appearance, and only the nurse who was the project contributor was aware of it. Other personnel, including the surgeon and anesthesiologist were not aware of the study groups. Allocation was concealed using sequentially numbered, sealed envelopes technique.

Postoperatively, all patients were connected to a PCA pump after being transferred to the post anesthesia care unit. The PCA solution contained 40 mg morphine in 80 ml normal saline. The PCA was set to administer a bolus dose of 0.5 ml with a lockout interval of 15 minutes and background infusion rate of 2 ml/min. Patients did not receive any antiemetic prophylaxis.

The primary outcome was effectiveness of IN ketamine and morphine PCA in decreasing pain intensity, compared to morphine PCA alone. All patients rated their pain, nausea, and headache intensity using VAS during 15, 30 and 60 minutes and 2, 6, and 12 hours, postoperatively. Evaluations of pain, nausea, and headache intensity as well as incidence of any side effects were recorded by anesthesiology resident who was blinded to the study groups. In addition, at the beginning of the study, the weight of all women in kilograms (using a standard scale with a minimum of clothing and without shoes) and their height in centimeters (without the shoe) was evaluated and the patients' body mass index (BMI) was calculated.

This information includes information such as age, level of education, number of children, place of residence, history of smoking, number of previous cesarean and vaginal deliveries, whether or not the pregnancy is desired, duration of surgery, total morphine consumption in 24 hours after surgery and time of first getting out of bed after surgery was evaluated and recorded. The anesthesia protocol was the same for all patients. We followed the Consolidated Standards of Reporting Trials (CONSORT) reporting guideline.

Sample size was calculated using GPower3.1 with the formula for calculation of samples of repeated measures, based on a presumed effect size of 0.2, a statistical power of 80%, and a type I error of 5%. The overall proper sample size was found to be 113 participants. We therefore recruited 120 patients to account for any dropouts.

**Statistical analysis**: We used the Shapiro-Wilk test to test whether data were normally distributed. Descriptive baseline characteristics for two groups' comparisons were tabulated as mean±SD, median (inter-quartile range) or as percentages. Comparing between two groups for categorical data were statistically analyzed using chi-square or Fisher-exact test and for continuous data were statistically analyzed using t-test and Mann-Whitney U test. Using General Linear Model (GLM) score of VAS between two groups were compared by repeated measurement ANOVA test. Time of evaluation was considered as within subject factor, intervention state (intranasal ketamine and placebo) as between subject factor. The time groups (interaction term) was considered as group differences (between intranasal ketamine and placebo) in their response over times. We tested Mauchley's Sphericity test for compound symmetry assumption. A p value of 0.05 or less was considered statistically significant. Data were analyzed using IBM SPSS statistics version 22 and Stata version 14.

**Ethical consideration:** The study was carried out in accordance with the principles of the Declaration of Helsinki. The current study was performed after the approval of the institutional ethics committee. The aim of the study was explained to the patients, and informed consent was obtained from all participants. Also, protocol of this study was registered in the Iranian Registry of Clinical Trials Database (IRCT20211011052726N1).

**Data sharing**: All relevant data and methodological detail pertaining to this study are available to any interested researchers upon reasonable request to corresponding author.

## Results

**Figure 1:**
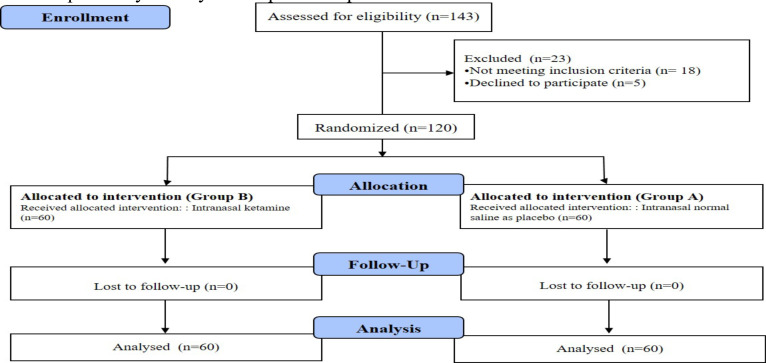
Flow diagram of the study

In this study, 143 women undergoing cesarean section referred to Imam Khomeini Hospital in Sari were evaluated in terms of response to the analgesic effects of intranasal ketamine, in terms of inclusion and exclusion criteria, as well as the satisfaction of participating in the study. 18 people did not meet the inclusion criteria and 5 people did not agree to participate in the study. 120 patients were randomly assigned to the two groups of intranasal ketamine and placebo in a 1: 1 ratio. Finally, all 120 subjects were evaluated in two groups of intervention and placebo (Figure 1).

The mean age of the patients was 29.28±4.3 years. The basic characteristics of patients were reported in Table 1. There was no significant difference between two groups in terms of demographic and clinical characteristics.

**Table 1:** Basic demographic and clinical characteristics of patients in the two groups

Variable		Group		*P-value*
			
		Intervention	Control	
**Age (year)**		30.72±3.14	29.83±4.36	0.46
**BMI**		31.64±3.21	32.11±16	0.23
**Dwelling place**	Village	22 (36.7%)	25 (41.7%)	0.58
	City	38 (63.3%)	35 (58.3%)	
**Education level**	Below high school diploma	0 (%)	3 (5%)	0.42
	High school diploma	18 (30%)	20 (33.3%)	
	Bachelor of Science or above	42 (70%)	37 (61.7%)	
**Unintended pregnancy**		39 (65%)	33 (55%)	0.26
**Cigarette smoking**		3 (5%)	1 (1.7%)	0.31
**Duration of surgery (mean±SD), minute**		69.32±8.25	71.95±9.55	0.35

**Figure 2:**
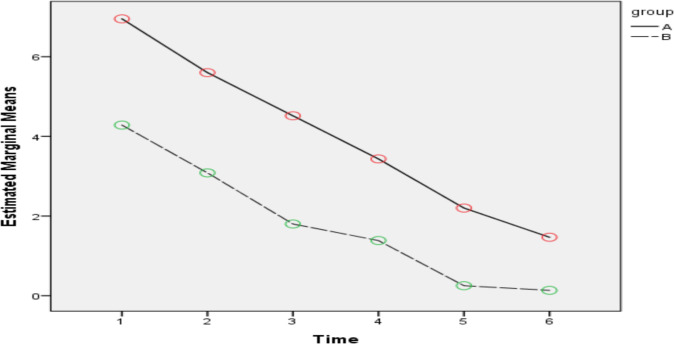
Changes in pain intensity in the two groups during the follow-up period.

**Figure 3:**
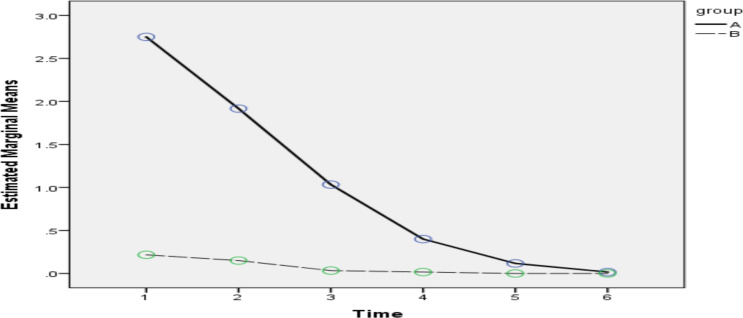
Changes in nausea intensity in the two groups during the follow-up period

Patients' postoperative pain and nausea intensity were assessed using VAS 15, 30 and 60 minutes and 2, 6, and 12 hours after surgery (Table 2 and 3, Figures 2 and 3). As shown in Table 2 and Figure 2, regardless of the study group, the trend of pain intensity changes was decreasing and these changes were statistically significant (time effect, *P<0.001*). Regardless of the time of study, the pain intensity in the placebo group was higher than the intervention and the observed difference was statistically significant (group effect, *P<0.001*). Although the trend of pain intensity changes in both groups was decreasing, the difference between the trend of pain intensity changes in the two groups was also statistically significant (interaction between the group and time effect, *P<0.001*). After controlling the effect of other variables in the GEE model, it was found that the level of pain intensity score of the intervention group compared to the placebo group was 1.97 units less (95% confidence interval: 1.73–2.22).

**Table 2:** The mean postoperative pain intensity in the two group

Variable		Group	

		Intervention (n=60)	Control (n=60)
VAS pain score, T1		4.28 (0.84)	6.95 (0.81)
VAS pain score, T2		3.8 (0.91)	5.61 (0.83)
VAS pain score, T3		1.8 (0.86)	4.52 (0.93)
VAS pain score, T4		1.38 (0.82)	3.43 (0.98)
VAS pain score, T5		0.25 (0.51)	2.20 (0.73)
VAS pain score, T6		0.13 (0.43)	1.47 (0.81)
*P*-value	Time effect	<0.001	
	Group effect	<0.001	
	Interaction effect	<0.001	

T1: 15 minutes after surgery; T2: 30 minutes after surgery; T3: 60 minutes after surgery; T4: 2 hours after surgery; T5: 6 hours after surgery; T6: 12 hours after surgery.

**Table 3:** The mean postoperative nausea intensity in the two groups

Variable		Group	

		Intervention (n=60)	Control (n=60)
VAS nausea score, T1		0.22 (0.73)	2.75 (1.18)
VAS nausea score, T2		0.15 (0.54)	1.92 (1.15)
VAS nausea score, T3		0.03 (0.18)	1.03 (0.95)
VAS nausea score, T4		0.02 (0.12)	0.43 (0.66)
VAS nausea score, T5		0 (0)	0.12 (0.37)
VAS nausea score, T6		0 (0)	0.02 (0.12)
	Time effect	<0.001	
*P*-value	Group effect	<0.001	
	Interaction effect	<0.001	

T1: 15 minutes after surgery; T2: 30 minutes after surgery; T3: 60 minutes after surgery; T4: 2 hours after surgery; T5: 6 hours after surgery; T6: 12 hours after surgery

As shown in Table 3 and Figure 3, regardless of the study group, the trend of changes in nausea severity was decreasing and these changes were statistically significant (time effect, *P<0.001*). Regardless of the time of study, the severity of nausea in the placebo group was higher than the intervention and the difference was statistically significant (group effect, *P<0.001*). Although the trend of nausea intensity changes in both groups is decreasing, but the slope of decreasing changes in the intervention group was higher than the placebo group and the difference between the trend of nausea intensity changes in the two groups was statistically significant (interaction effect between group and time, *P<0.001*). After controlling the effect of other variables in the GEE model, it was found that the level of nausea score in the intervention group compared to the placebo group was 0.37 units less (95% confidence interval: 0.24–0.51).

While no person in the intervention group had vomiting, but in the placebo group, 5 people (8.3%) had this complication and the difference in the ratio of vomiting in the two groups was statistically significant (*P=0.02*). The median (first quarter-third quarter) morphine consumption in the placebo and intervention groups was 8 (6–8) and 2 (2-4) mg, respectively, and the difference was statistically significant (*P<0.001*).

## Discussion

Postoperative analgesia after CS is challenging since we need not only to consider maternal comfort, but the anesthetic technique should also have no adverse drug reactions on the newborn. The results of the present study showed that intranasal administration of ketamine significantly reduced the pain intensity of patients after CS and the amount of morphine consumption in the postoperative period. Very limited studies have investigated the effect of intranasal ketamine for reducing the pain intensity after surgery, especially CS. Most studies that have been performed to evaluate the analgesic effect of intranasal ketamine have been performed in the emergency department. In a study by El-Halwagy et al., intranasal administration of ketamine at a dose of 0.5 mg/kg, compared with intramuscular pethidine, significantly reduced the pain intensity of patients after CS (28), which is in line with the findings of the present study.

In another study, it was shown that intranasal ketamine (1.5 mg/kg) and intranasal fentanyl were associated with a significant reduction in pain intensity after tonsillectomy in children. There was no statistically significant difference between the two groups in the postoperative period, although the incidence of sedative effects was higher in the intranasal ketamine group than in the other group (21).

Another study in patients undergoing spinal surgery showed that intranasal administration of S-ketamine (6 mg) as well as intranasal midazolam, compared with morphine administration using a PCA pump, was significantly associated with lower postoperative pain intensity. In general, there was no statistically significant difference in the incidence of complications in the three groups, although the incidence of nystagmus in the S-ketamine group and dry mouth in patients receiving morphine were slightly higher than the other groups (22).

In their study, Frey et al. showed that intranasal ketamine use significantly reduced the severity of pain in children with traumatic limb injuries referred to the emergency department compared to intranasal fentanyl (29). The positive effects of intranasal ketamine, compared with intravenous morphine, have also been shown to significantly reduce pain intensity in patients with bone fractures (30). In a study by Shimonovich et al. it was also shown that intranasal ketamine has similar analgesic effects similar to intravenous and intramuscular morphine in patients with moderate to severe acute traumatic pain admitted to the emergency department (31). Also it has been shown that early administration of intranasal ketamine during triage is associated with less pain intensity and less need for opioid and non-opioid drugs in patients with acute pain due to limb trauma (32).

In a study conducted by Quinn et al. which aimed to determine and compare the effect of intramuscular ketamine and fentanyl on moderate and severe pain in children referred to the emergency department, the results showed that in terms of pain intensity in two groups, there was no statistically significant difference 20 minutes after drug administration. However, the incidence of side effects in the ketamine group was significantly higher than the fentanyl group (33). In a study, which examined and compared the effect of intranasal ketamine and intravenous morphine on pain intensity in patients with renal colic referred to the emergency department, the results indicated that ketamine was found to be less effective than fentanyl in controlling renal colic-induced pain, and to be associated with a higher prevalence of side-effects (34). In a recent systematic review and meta-analysis to evaluate the effect and safety of intranasal ketamine on acute pain in adults referred to the emergency department, the findings showed the positive effects of intranasal ketamine in reducing acute pain in patients referred to the emergency department. It was recommended that this method can be used instead of prescribing intravenous analgesics in these patients. In terms of side effects, it was also shown that patients receiving intranasal ketamine experienced only mild side effects (35). Also, some published studies evaluate and confirmed the efficacy of intranasal ketamine for pediatric sedation (36–37).

Intranasal ketamine has been shown to have 45% bioavailability, which is higher than other methods of prescribing this drug. The clinical effects of ketamine can probably be explained by the absorption of the drug through the nasal mucosa, which allows it to act on the brain without first-pass metabolism. In a study evaluating the efficacy and pharmacokinetics of ketamine, it was shown that within 2 minutes after intranasal administration, ketamine was measurable in the blood at concentrations much higher than its less active metabolite, norectamine (38). There is also little evidence in the literature about significant side effects of intranasal ketamine. One study found that long-term intranasal ketamine use (four times an hour over a 1-month period) was associated with anosmia in women with severe cancer pain (39).

In previous studies, drowsiness and dizziness have been mentioned as common side effects, which are usually mild or moderate, as well as temporary and transient and disappeared within 7–10 minutes (23, 31). There were some limitations to our study. First, we did not assess the possible preoperative confounding factors for pain after CS. Second, the observation period was the initial 12 hours, and we recommend that future studies lengthen the observation time to 48 h or even longer. Third, this is a single-center study, which may limit the generalizability of the findings. In conclusion, based on the findings of this study, it seems that the use of intranasal ketamine at a dose of 1 mg/kg, can be an effective, tolerable and safe method in reducing pain intensity and also the need for opioid use after CS.
